# Carotid sinus massage in clinical practice

**DOI:** 10.1093/europace/euaf058

**Published:** 2025-03-18

**Authors:** Sebastian Stec, Marta Kornaszewska

**Affiliations:** Department of Cardiac Surgery and Transplantation, Hybrid Electrophysiology and Arrhythmia Management Program, National Medical Institute of the Ministry of the Interior and Administration, Warsaw, Poland; Private Cardiology Practice, Kleczany 238, 33-395 Chelmiec, Poland

We read with great interest the article by de Lange *et al*.^1^ on carotid sinus massage (CSM) and that CSM requires ‘re-implementation’ in clinical practice in patients with syncope, cardiovascular autonomic dysfunction (CVAD), and arrhythmias. We would like to comment on several aspects.

First, while current data do not support the use of CSM for diagnosing carotid sinus syndrome, its relevance has increased in the post-COVID era, particularly among young patients with neurological or oncological conditions. Carotid sinus massage should be included in standard protocols for CVAD and syncope using cardiovascular autonomic testing (CAT). Torabi *et al*.^2^ showed that complex syncope can be diagnosed non-invasively using CAT, enabling tailored treatments. This is crucial, especially in the era of innovative and alternative interventions like cardioneuroablation (CNA), sinus node sparing hybrid ablation, vagal nerve stimulation, cardiac sympathetic denervation, conduction system pacing, or renal denervation.^3–9^

Avoiding CSM due to carotid bruise, peripheral artery disease risk, patient reluctance, or vulnerable plaque in the carotid artery should be documented in clinical reports. We emphasize that whether CSM was performed should be clearly recorded in medical records, aligning with shared decision-making principles.^9^

Secondly, data on the reproducibility of CSM in both elective and urgent settings are limited. Research comparing single or multiple uses of CSM with techniques like the Valsalva manoeuvre is scarce, particularly regarding paroxysmal supraventricular tachycardia termination and validation of the effectiveness of interventional procedures.

Thirdly, continuous beat-to-beat blood pressure (BP) monitoring improves diagnostic accuracy over intermittent methods, which may miss transient BP drops, underestimating vasodepressor syncope.^7^ Cuffless BP technologies offer real-time, non-invasive monitoring, enhancing syncope evaluation.^10^ Integrating these innovations with CSM could refine autonomic dysfunction detection and improve syncope management.^9^ Future studies should validate the diagnostic accuracy of this approach.

Fourthly, ‘heart rate’ monitoring with a 3 s pause may be too general and does not fully reflect the impact of CSM on clinical decision-making today and in the near future. Instead of just ‘heart rate’, heart rhythm and conduction properties should be validated, including P wave morphology, PR interval, atrioventricular block (AVB), QRS complex morphology, QT interval changes, and repolarization parameters. Therefore, at least limb lead II or, ideally, a standard 12-lead electrocardiogram will be of great value, especially with the rise of CNA, to guide strategy selection and efficacy assessment after sinoatrial or atrioventricular node denervation.^4,5^ Furthermore, CSM could help monitor CNA’s effectiveness if it consistently induces severe sinus bradycardia, sinus arrest, prolonged PR interval, or advanced AVB.

Finally, we appreciate the authors’ discussion on electrocardiographic monitoring during CSM and the need for detailed conduction analysis. While CNA is frequently referenced in our letter as an emerging therapeutic option, we acknowledge that current evidence is based on observational studies rather than randomized controlled trials (RCTs). Given the significant placebo effect observed in pacing trials for reflex syncope, such as the VPS II and VASIS-PM studies, further RCTs are needed to establish CNA’s definitive role. Until then, its application should be considered cautiously within an individualized treatment approach.
